# eHealth Interventions for HIV Prevention in High-Risk Men Who Have Sex With Men: A Systematic Review

**DOI:** 10.2196/jmir.3393

**Published:** 2014-05-26

**Authors:** Rebecca Schnall, Jasmine Travers, Marlene Rojas, Alex Carballo-Diéguez

**Affiliations:** ^1^Columbia UniversitySchool of NursingNew York, NYUnited States; ^2^Columbia UniversityHIV Center for Clinical and Behavioral StudiesNew York State Psychiatric InstituteNew York, NYUnited States

**Keywords:** HIV prevention, eHealth, high-risk MSM, HIV testing, HIV risk behaviors, SMS, Internet

## Abstract

**Background:**

While the human immunodeficiency virus (HIV) incidence rate has remained steady in most groups, the overall incidence of HIV among men who have sex with men (MSM) has been steadily increasing in the United States. eHealth is a platform for health behavior change interventions and provides new opportunities for the delivery of HIV prevention messages.

**Objective:**

The purpose of this systematic review was to examine the use of eHealth interventions for HIV prevention in high-risk MSM.

**Methods:**

We systematically searched PubMed, OVID, ISI Web of Knowledge, Google Scholar, and Google for articles and grey literature reporting the original results of any studies related to HIV prevention in MSM and developed a standard data collection form to extract information on study characteristics and outcome data.

**Results:**

In total, 13 articles met the inclusion criteria, of which five articles targeted HIV testing behaviors and eight focused on decreasing HIV risk behaviors. Interventions included Web-based education modules, text messaging (SMS, short message service), chat rooms, and social networking. The methodological quality of articles ranged from 49.4-94.6%. Wide variation in the interventions meant synthesis of the results using meta-analysis would not be appropriate.

**Conclusions:**

This review shows evidence that eHealth for HIV prevention in high-risk MSM has the potential to be effective in the short term for reducing HIV risk behaviors and increasing testing rates. Given that many of these studies were short term and had other limitations, but showed strong preliminary evidence of improving outcomes, additional work needs to rigorously assess the use of eHealth strategies for HIV prevention in high-risk MSM.

## Introduction

Men who have sex with men (MSM) are the population most heavily affected by infection with human immunodeficiency virus (HIV) [1]. The rate of a new HIV diagnosis among MSM is more than 40 times that of women and more than 44 times that of other men [2]. In 2010, male-to-male sex remained the largest HIV transmission category in the United States and the only one associated with an increasing number of HIV/ acquired immunodeficiency syndrome (AIDS) diagnoses [3]. Although MSM represent about 7% of the male population in the United States, they account for 78% of the new HIV infections among males, reinforcing the need for intensive HIV prevention services and testing campaigns [4].

The number of new HIV infections among MSM increased 12% from 2008-2010, with a 22% increase among MSM aged 13-24 years. Notably, young African American MSM account for a disproportionate number of new HIV cases in the United States. There were more new HIV infections (54%) among young African American MSM (aged 13-29 years) than any other racial or ethnic age group of MSM [5], which is nearly twice that of young white MSM and more than twice that of young Hispanic/Latino MSM [6]. Increases in the number of HIV positive individuals in this group suggest that risky sexual behavior has risen despite advances made in testing, prevention, and treatment [5].

While many HIV prevention interventions have been delivered face to face, the emergence of eHealth as a platform for health behavior change provides new opportunities for developing HIV prevention strategies [7]. eHealth is a generic term that applies to an increasingly large number of interventions that are delivered electronically. eHealth can include Web-based tools including videos, games, chat rooms, social networking sites as well as text messaging (SMS, short message service), and email [8]. Across a wide range of diseases and health behaviors, eHealth interventions are successful in promoting changes in behavior, self-efficacy, knowledge, and clinical outcomes. eHealth interventions have been developed to prevent obesity [9,10], treat alcohol abuse [11], promote smoking cessation [12], and encourage nutritious eating [13].

The Internet is an important delivery method for eHealth tools. As access to the Internet increases, Americans’ willingness to use the Internet as a source of health information has proliferated, suggesting Web-based interventions are an important modality for health behavior change interventions [14]. Online interventions can be extremely convenient for users as they are accessible from anywhere that there is connection to the Internet and can be used in a private setting, which can also improve accessibility [15]. In a recent systematic review, Guse et al evaluated the impact of digital media-based interventions on the sexual health knowledge, attitudes, and/or behaviors of adolescents aged 13-24 years. Two interventions significantly delayed initiation of sex, and one was successful in encouraging users of a social networking site to remove sex references from their public profile. Seven interventions significantly influenced psychosocial outcomes such as condom self-efficacy and abstinence attitudes, and six studies increased knowledge of HIV, sexually transmitted infections, or pregnancy [16].

A growing number of eHealth HIV prevention interventions have been developed for MSM [17-20]. eHealth interventions are particularly relevant for this high-risk population because of the privacy feature they provide. A user can privately access them without the fear of stigma, which highly affects the MSM community [21]. As the evidence-base on eHealth HIV prevention interventions grows, there is also a need to systematically evaluate the efficacy and feasibility of the existing interventions specific to the MSM population.

## Methods

### Identification of Studies

We searched articles published from January 2000 to April 2014 in the following electronic databases: PubMed, PsycINFO, Embase, ISI Web of Knowledge, Google Scholar, and Google for grey literature of US and international studies. We visually scanned the reference lists of retrieved documents to identify additional relevant manuscripts. Our search terms included HIV, online, mobile technology, AIDS, technology, electronic health, eHealth, chat room, social networking, mobile applications, mobile health applications, mobile phone, mHealth, text messaging, telemedicine, HIV treatment, PLWH, reminder systems, information systems, Computers, Handheld/ or Cellular Phone/ or mobile applications, HIV/ or HIV.mp or HIV Infections/; Cellular Phone/ or mobile application.mp; HIV/, HIV Infections/ or PLWH.mp, HIV infection, intervention, mobile applications, and mobile HIV applications.

### Inclusion Criteria

Included studies had to (1) focus on an eHealth intervention only and could not use eHealth solely as a recruitment or data collection tool, (2) focus on HIV prevention or testing and not on HIV care, (3) be published in English, (4) be published between January 2000 and April 2014, (5) be quasi-experimental or a randomized controlled trial (RCT), (6) have a behavioral outcome measure, and (7) focus on adult MSM. We did not include adolescent studies in our review since a recent systematic review was published [16].

### Assessing Study Quality

A quality assessment tool (Table 1) for evaluating HIV prevention interventions was created based on the previously published efficacy criteria developed by the Center for Disease Control and Prevention’s HIV/AIDS Prevention Research Branch [22,23]. Papers were scored in each of seven quality domains, and a final total score was calculated as a percentage of possible applicable points. The domains were representativeness, bias and confounding, description of the intervention, outcomes and follow-up, statistical analysis, strength of evidence, and group equivalence. Each of the seven quality domains was given equal weight.

**Table 1:** HIV prevention intervention quality assessment tool.

	Completely adequate (%)	Partially adequate (%)		Inadequate, not stated, or impossible to tell (%)
Representativeness	All key characteristics of study population described (50)	Some key characteristics described (25)		Minimal to no description of key characteristics and inclusion/exclusion criteria (0)
	Detailed inclusion/exclusion criteria described (50)	Some description of inclusion/exclusion criteria (25)		
Bias and confounding	Study population corresponded to larger population in all key factors (25)	Sample population differed in some minor factors to larger population (12.5)		Sample population differed in several key factors to larger population (0)
	Equivalent outcome assessment (25)	Minor differences in outcome assessment (12.5)		Major differences in outcome assessment (0)
	Study accounted for confounding interventions with respect to effectiveness of intervention (25)	Study only partially accounted for confounding interventions with respect to effectiveness of intervention (12.5)		Study did not account for confounding interventions with respect to effectiveness of intervention (0)
	Compliance rate >80% (25)	Compliance rate between 80-50% (16.7)		Compliance rate <50% (8.3)
Description of intervention	Protocol could be replicated given description of intervention and /or monitoring (100)	Some minor details excluded from explanation of intervention and/or monitoring (66.7)		No details given in description of intervention and monitoring (0)
		Some major details excluded from explanation of intervention and/or monitoring (33.3)		
Outcomes and follow-up	Outcome assessment procedure clearly defined (50)	Outcome assessment procedure somewhat defined (25)		Outcome assessment procedure not defined (0)
	Groups equivalent in attrition (50)	Some difference in attrition (25)		Major difference in attrition (0)
Statistical analysis	Statistical methods fully described and appropriate (50)	Statistical methods partially described and appropriate (25)		Statistical methods not described or absent (0)
	Tests addressed differences between groups and variability (50)	Tests addressed some differences between groups and variability (25)		Did not address differences between groups and variability (0)
Strength of evidence	Significant positive intervention effects (100)	Significant effect but not in the stated relevant outcome measure (50)		No significant intervention effect (0)
	Positive and statistically significant (*P*≤.05) intervention effect in ≥1 relevant outcome measure			
Group equivalence	Meets all 4 criteria (100)	Meets 3 criteria (75)		Meets no criteria (0)
	1. Include one or more separate control or comparison study groups.	Meets 2 criteria (50)		
	2. Include clear description of study group comparability.	Meets 1 criteria (25)		
	3. Include clear description of randomization method used or rationale for not using randomization technique in instances when it is not feasible			
	4. Include appropriate statistical controls when equivalence is not achieved			

### Data Extraction

**Figure 1:**
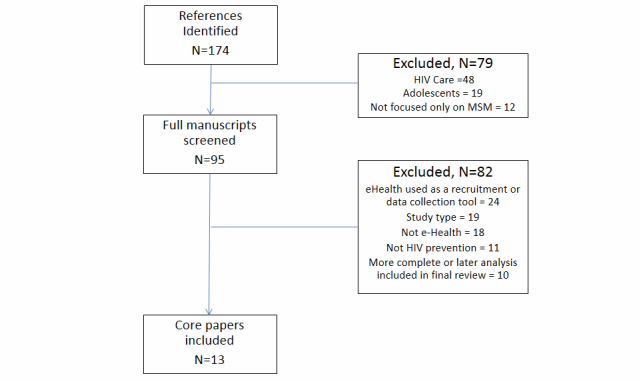
Screening process flowchart.

Figure 1 summarizes the search results and the outcome of the screening process. The search identified 174 unique papers. These papers were independently appraised by 2 authors with no blinding to the authorship of the papers. Following our appraisal of the abstracts, we excluded articles that were focused on HIV care for persons living with HIV, 19 articles focused on adolescents, and 12 articles that did not solely focus on MSM (eg, women, all men, mixed genders). We reviewed the full manuscript of the remaining 95 articles. Of these, we excluded 10 articles because there was a later published article that was more recent and/or of higher quality. We also excluded 19 other articles because they used a qualitative research design or did not have a behavioral outcome (see Figure 1 for further details of articles that were excluded).

Data were extracted based on objectives, study design, sample size, type and duration of interventions, outcome measures reported, and findings. To further characterize the intervention, we abstracted the theoretical framework used to guide the intervention design, if reported. Data were also abstracted according to country.

### Characteristics of Evidence

A total of 13 articles met the inclusion criteria. The articles were published between 2008 and 2013. Table 2 describes each paper by study design, eHealth strategy, theoretical framework, length of intervention and follow-up, study population, results, and quality rating [24-36]. The total sample in each study ranged from 52 [24] to 3092 men [25]. Nine of the studies were conducted in the United States, and the remaining studies were conducted in Peru (n=1), Australia (n=1), Taiwan (n=1), and Hong Kong (n=1).

Each of the studies had different interventions. Interventions were not clearly described in approximately 46% of the studies. The length of the intervention period ranged from about 15 minutes [25] to 6 months [26-29]. Consistent with inclusion criteria, all studies targeted MSM, with one study [30] focusing on rural MSM and one study [24] focusing on methamphetamine users. Outcome measures included condom use, HIV testing rates, and sexual risk behaviors. eHealth strategies included Web-based videos and education modules (7 studies), text messaging (3 studies), chat room intervention (1 study), and social networking (2 studies). Details of the interventions are described below. A majority (69%) of the interventions were guided by a theoretical framework including the Information-Motivation-Behavioral Skills Model (IMB), the Health Belief Model, Stages of Change, Social Learning Theory, Social Cognition and Developmental Theory, and the Sexual Health Model. The remaining interventions were not guided by theoretical frameworks, or details were not provided in the article. Eight of the studies used an RCT and five of the studies were quasi-experimental. The methodological quality of the available evidence varied, and none of the included studies fulfilled all of the criteria, with quality scores ranging from 49.4-94.6%.

**Table 2:** Existing studies of eHealth HIV prevention interventions for adult MSM.

Study	Study design	eHealth Strategy	Length of study	Study population	Results	Mean quality score (range)
Blas, 2010 [31]	RCT	Web-based Intervention	Mean of 125.5 days of observation	Intervention (N=239); Comparison (N=220)	Increased HIV testing rates	76.79% (50-100)
Bourne, 2011 [26]	Pre-post test design	SMS reminders	SMS reminders every month for 3-6 months	Intervention (N=714); Control (N=1084)	Increased HIV re-testing rates	51.14 (25-100)
Bowen, 2008 [30]	Pre-Post study	Web-based education modules	Mean 19.39 days (SD 7.33 days)	Rural MSM (N=475)	Decrease high-risk sexual risk behaviors;	89.89 (50-100)
Carpenter, 2010 [32]	RCT	Web-based skills training and motivational intervention	Intervention 1.5-2 h; 3-month follow up	MSM (N=112)	Reduction in high-risk HIV behavior	64.89 (0-100)
Christensen, 2013 [33]	RCT	Virtual Simulation Intervention	3-month follow-up questionnaire	Intervention (N=437) Control (N=484)	Shame reduction; shame reduction as a predictor of UAI^a^	75 (33.3-100)
Hirshfield, 2012 [25]	RCT	Web-based media intervention (prevention videos & webpage)	Baseline survey, Intervention 60 day follow up	Intervention (N=2483) Control (N=609)	More likely to disclose HIV status to partners; less likely to report UAI	89.89 (54.2-100)
Ko, 2013 [27]	Quasi-Experimental, Non-Equivalent control	Web-based peer leader intervention	Baseline survey, 6-month intervention, follow-up survey	Intervention (N=499); Comparison (N=538)	Increased HIV testing, reduced UAI	60.11 (33-100)
Lau, 2008 [28]	RCT	Web-based educational tool	6-month study period	Intervention (N=140); Control (N=140)	Efficacy of the intervention was not supported	65.49 (0-100)
Mustanski, 2013 [34]	RCT	Web-based media intervention	12-wk study period	Intervention (N=50); Control (N=52)	Decrease sexual risk behavior	94.64 (75-100)
Reback, 2012 [24]	Pre-post test design	Text Messaging	2-wk intervention	Meth-using MSM (N=52)	Decreased frequency of methamphetamine use; Decrease high-risk sexual behaviors.	83.93 (50-100)
Rhodes, 2011 [29]	Single-group pretest-post-test design	Chat Rooms	6-month implementation phase; 1-month follow-up	MSM (N=346 [pretest], 315 [posttest])	Increased HIV testing rates	64.89 (25-100)
Rosser, 2010 [35]	RCT	Interactive Website	3-wk intervention	MSM (N=650)	Reduction in risk behavior	49.41 (25-66)
Young, 2013 [36]	RCT	Web Based, Peer leader led groups	12-wk intervention; 12-wk follow-up	112 MSM Intervention N=55 Control N=57	Increased requests for an HIV home test	80.36 (50-100)

^a^UAI: unprotected anal intercourse

### Web-Based Videos and Education Modules (k=7)

Two studies used videos for educating high-risk MSM. In a study conducted in Peru, 5-minute videos were created using the Health Belief Model and Stages of Change Theory to encourage MSM to get tested for HIV. The videos incorporated ways to overcome eight reasons why MSM do not get tested for HIV (eg, fear or lack of confidentiality) [31]. In another study, informed by the Social Learning Theory and Social Cognition and Developmental Theory, five study conditions were compared using an RCT and included a (1) dramatic video, (2) documentary video, (3) both dramatic and documentary videos, (4) prevention webpage, and (5) control (received no intervention) [25]. “The Morning After” is a 9-minute video drama [37] that was designed to promote critical thinking about HIV risk and features 3 gay male friends, one of whom thinks he had unprotected sex with an HIV-positive man while intoxicated and seeks advice from friends.“*Talking About HIV”* is a 5-minute documentary video created with footage from the documentary, “Meth” [38]. In the 60 days after the intervention, men in the pooled video group were significantly more likely than men in the control group to report full disclosure with their last sexual partner (OR 1.32, 95% CI 1.01-1.74). HIV positive men in this group were also significantly more likely to reduce unprotected anal intercourse (UAI; OR 0.38, 95% CI 0.20-0.67) and serodiscordant UAI (OR 0.53, 95% CI 0.28-0.96) at follow-up. Findings from this study suggest that a brief digital media intervention can decrease sexual risk behaviors and increase HIV disclosure to potential sexual partners [25].

One study developed and tested multicomponent Internet sites that targeted high-risk sexual behaviors. The intervention, Sexpulse, was a multifaceted Internet intervention that targeted men who use the Internet to seek sex with men and was informed by the Sexual Health Model. Sexpulse was designed by a multidisciplinary team of health professionals, computer scientists, and e-learning specialists and had the following components: a risk assessment tool, an online chat simulation, and virtual peers. Use of the system successfully reduced high-risk sexual behavior in study participants [35]. Carpenter et al developed an Internet site [39] based on the Information Motivation Behavioral Skills theory of HIV risk reduction, which included risk assessment and feedback, motivational exercises, skills training, and education. The format of the material was designed to engage younger MSM, including those from minority groups. Both the intervention and control groups demonstrated reductions in high-risk sexual activity; the intervention group showed greater reductions with the riskiest partners [32].

Keep It Up! (KIU!) was an online, interactive HIV prevention program. The IMB model of HIV risk behavior change was used to guide the development of the KIU! intervention. It has 7 modules completed across 3 sessions that were done at least 24 hours apart and takes about 2 hours to complete. Keep It Up! was designed to be delivered to young MSM upon receiving an HIV negative test result. In an RCT, the participants in the intervention arm had a significantly lower rate of unprotected anal sex acts at the 12-week follow-up [34].

Socially Optimized Learning in Virtual Environments (SOLVE) is a downloadable simulation video game designed to simulate and immerse high-risk young adult MSM in affectively charged risky situations. This intervention was informed by the Theory of Planned Behavior, and Social Cognitive Theory. Christensen et al tested this intervention compared to a wait list control condition in an online RCT. After 3 months, participants in the SOLVE treatment condition reported greater reductions in shame. The direct effect on risky sexual behavior at follow-up was not significant [33].

Finally one study developed and tested a Web-based education module tailored to the information needs of MSM residing in rural areas. There were two 20-minute education sessions that participants watched 6 months apart. Each session consisted of three modules focused on the concepts in the IMB model. Post-intervention behavior change included reduced anal sex and significant increases in condom use [30].

### Text Messages or Short Messaging Service and Email Messaging (k=3)

Three studies in our review used text messaging or short messaging service (SMS) as an intervention; two studies used it to increase HIV testing rates and one study to reduce high risk behaviors. The two studies that targeted increasing HIV testing rates were conducted outside of the United States. In one of the SMS studies set in Australia, clinicians sent reminders to patients who had previously come to a sexual health clinic to come back for follow-up testing. SMS reminders increased HIV re-testing rates after 9 months [26]. In the Project Tech Support study, participants received 1-3 social support and health education text messages per day for 2 weeks. The goal of the messages was to reduce methamphetamine use and high-risk sexual behaviors. A total of 400 text messages were developed for this study based on the behavioral change theories of Social Support Theory, the Health Belief Model, and Social Cognitive Theory. Participants reported a significant decrease in methamphetamine use and reductions in high-risk sexual behaviors [24]. In the study conducted in Hong Kong, email messages relating to prevention of STI (sexually transmitted infections) and HIV were sent to participants on a biweekly basis [28]. The contents of the emails covered areas of information and discussion about modes of HIV transmission, correct condom use, HIV testing, “relationships & love”, and the relationship between drugs and sex. The goal of this intervention was to reduce HIV risk-related behaviors; however, there were no significant findings.

### Chat Room Intervention (k=1)

One study used a chat room intervention named CyBER/testing, informed by the Natural Helping Theory, in which an interventionist entered the chat room from 9 a.m.-5 p.m., Monday to Friday [29]. Few details about the chat room were included in the study to protect the participants who still use the site. The chat room was designed for social and sexual networking among MSM. Every 30 minutes, the interventionist would post information about HIV testing. More specifically, he answered questions about testing processes and locations, referred chatters to other resources, explained HIV infection, and provided information about resources for those who are seropositive (including medical resources and AIDS drug assistance). He would also respond to chat room members who sent him “instant messages”. The intervention significantly increased self-reported HIV testing among chatters overall.

### Social Networking Intervention (k=2)

In the HOPE study, social network sites were used for the delivery of HIV prevention information; 16 peer leaders were randomly assigned to deliver information about HIV (intervention) or general health (control) via Facebook groups for over 12 weeks. Participants randomized to the HIV prevention information group were significantly more likely to request an HIV testing kit than control group participants [36]. There were sparse data on returned tests and follow-up test results indicating that even though this intervention influenced participants’ decision to request an HIV test, it did not necessarily impact actual testing behaviors [36].

In another social networking intervention study, Internet popular opinion leaders (iPOL) were used to disseminate HIV prevention information via popular social networking sites [27]. At the 6-month follow-up after the intervention was conducted, MSM who visited the intervention website were more likely to have been tested for HIV (*P*<.001) and consistently use condoms during anal sex with online sex partners than those using the control website (34.15% versus 26.19%, *P*=.004). This study used a non-equivalent quasi-experimental design. There were additional flaws in the study design that limit the evidence of the use of this intervention for improving HIV prevention behaviors including contamination between study groups and self-report of HIV testing and risk behaviors.

## Discussion

### Principal Findings

This review of eHealth interventions for HIV prevention among adult MSM has drawn together the evidence base specific to behavioral interventions for MSM and found evidence for eHealth interventions being associated with reductions in high risk behaviors and increases in HIV testing rates. Nonetheless, the studies that demonstrated a decrease in sexual risk behavior had different study designs and outcome measures that make it difficult to synthesize the evidence.

Only one US study in our review solely focused on HIV testing as an outcome measure. Given that the US National HIV/AIDS Strategy has established a goal of increasing the awareness of HIV status in the US population from 79% to 90% by 2015, HIV testing is an important HIV prevention measure. In fact, current recommendations are to repeat HIV testing every 3-6 months for high-risk MSM [40]. Thus, there is a need to develop and test eHealth interventions targeted to improve HIV testing rates in high-risk MSM. Given that most of the eHealth intervention studies conducted abroad targeted HIV testing behaviors, future work in the United States should focus on the lessons learned from those studies.

Bourne et al found that SMS can be used to increase HIV testing rates in high-risk MSM [26]. In a single study by Blas et al [31], the use of an online video-based intervention was shown to increase HIV testing in high-risk MSM. Given the growing HIV epidemic in high-risk MSM in the United States and the need to increase HIV testing, both online video-based interventions and certainly SMS should be employed as strategies to increase HIV testing rates. The use of SMS for improving HIV testing rates was evidenced in studies outside of the United States showing promise for its continued use both abroad and in the United States.

From the results presented above, we can infer that eHealth interventions reduce risky sexual behaviors and increase HIV testing. This review has provided evidence that eHealth interventions have the potential for promoting HIV prevention behaviors in adult MSM. Even so, there are a number of limitations in many of the studies we reviewed. For example, in the study conducted by Reback et al (2012) [24], there was no control group, the intervention group was quite small, and most of the study participants were unemployed, which is not necessarily representative of the MSM population. The study intervention was staff intensive and used two-way pagers that no longer exist, limiting the potential for harnessing this technology for future intervention study.

In another study, Young et al (2013) [36] had planned 7 clusters per study arm but ended up with only 2. Recruitment appeared to have been difficult; they used only Facebook, no sex websites (which could have been more efficient to reach people having high risk behavior). To assess their outcome measure, they used HIV tests that needed to be sent to a laboratory. Participants could have used an HIV home test, which may have reduced some of the access barriers. This review highlights the need for the collection of rigorous data measures for understanding outcomes.

Moreover, there is a need for long-term (12 months) follow-up data after the completion of eHealth HIV prevention interventions. In our review, only 1 study assessed the long-term effects (12 months) of the eHealth intervention and found that it did not have a long-term effect on reducing sexual risk behaviors [35], perhaps because this was not a long-term intervention. Since eHealth interventions appear potentially useful for reducing HIV risk behaviors and increasing HIV testing rates, future research should focus on establishing long-term effectiveness as well as comparing the effectiveness of different interventions.

### Limitations

Several limitations of this review should be considered when interpreting the findings. The potential heterogeneity of interventions and outcomes are important to note and make the synthesis of the evidence from these studies challenging. Notably, even though we attempted to be as inclusive as possible, our searches may have excluded relevant studies from this systematic review that did not meet our search word criteria, and/or we excluded conference abstracts that met this review’s criteria but were not peer-reviewed articles.

### Conclusions

Our results have important implications for the use of eHealth interventions for HIV prevention in MSM. This review demonstrates eHealth interventions appear potentially useful for reducing HIV risk behavior and increasing HIV testing rates. The detailed data across the studies allows us to comprehensively identify and describe elements that are essential to the effectiveness of eHealth interventions for promoting HIV prevention among adult MSM. Given the limitations of many of these studies as well as the potential for eHealth to transform health behaviors, additional work needs to rigorously assess the use of eHealth strategies for HIV prevention in high-risk MSM. Future work is needed that employs these interventions in longer and larger trials and to assess their efficacy in improving outcomes.

## Abbreviations

AIDS: acquired immunodeficiency syndrome
CDC: Centers for Disease Control and Prevention
HIV: human immunodeficiency virus
IMB: Information-Motivation-Behavioral Skills Model
MSM: men who have sex with men
RCT: randomized controlled trial
SMS: short message service
UAI: unprotected anal intercourse

## References

[1] Hall HI, Song R, Rhodes P, Prejean J, An Q, Lee LM, Karon J, Brookmeyer R, Kaplan EH, McKenna MT, Janssen RS, HIV Incidence Surveillance Group (2008). Estimation of HIV incidence in the United States. JAMA.

[2] Centers for Disease Control and Prevention (2010). CDC Analysis Provides New Look at Disproportionate Impact of HIV and Syphilis Among US Gay and Bisexual Men.

[3] Centers for Disease Control and Prevention Today's HIV/AIDS Epidemic.

[4] (2011). Center for Disease Control.

[5] Centers for Disease Control and Prevention (CDC) (2012). Vital signs: HIV infection, testing, and risk behaviors among youths - United States. MMWR Morb Mortal Wkly Rep.

[6] Centers for Disease Control and Prevention (CDC) (2008). Trends in HIV/AIDS diagnoses among men who have sex with men--33 states, 2001-2006. MMWR Morb Mortal Wkly Rep.

[7] Noar SM (2012). eHealth Applications: Promising Strategies for Behavior Change.

[8] Bennett GG, Glasgow RE (2009). The delivery of public health interventions via the Internet: actualizing their potential. Annu Rev Public Health.

[9] Glasgow RE, Boles SM, McKay HG, Feil EG, Barrera M (2003). The D-Net diabetes self-management program: long-term implementation, outcomes, and generalization results. Prev Med.

[10] McKay HG, King D, Eakin EG, Seeley JR, Glasgow RE (2001). The diabetes network internet-based physical activity intervention: a randomized pilot study. Diabetes Care.

[11] Saitz R, Helmuth ED, Aromaa SE, Guard A, Belanger M, Rosenbloom DL (2004). Web-based screening and brief intervention for the spectrum of alcohol problems. Prev Med.

[12] Civljak MS, Stead LF, Hartmann-Boyce J, Sheikh A, Car J (2013). Internet-based interventions for smoking cessation. Cochrane Database Syst Rev.

[13] Alexander GL, McClure JB, Calvi JH, Divine GW, Stopponi MA, Rolnick SJ, Heimendinger J, Tolsma DD, Resnicow K, Campbell MK, Strecher VJ, Johnson CC, MENU Choices Team (2010). A randomized clinical trial evaluating online interventions to improve fruit and vegetable consumption. Am J Public Health.

[14] McMullan M (2006). Patients using the Internet to obtain health information: how this affects the patient-health professional relationship. Patient Educ Couns.

[15] Binik Y (2001). Sexuality and the internet: Lots of hyp(otheses)—only a little data. Journal of Sex Research.

[16] Guse K, Levine D, Martins S, Lira A, Gaarde J, Westmorland W, Gilliam M (2012). Interventions using new digital media to improve adolescent sexual health: a systematic review. J Adolesc Health.

[17] Moskowitz DA, Melton D, Owczarzak J (2009). PowerON: the use of instant message counseling and the Internet to facilitate HIV/STD education and prevention. Patient Educ Couns.

[18] Wei C, Lim SH, Guadamuz TE, Koe S (2013). Virtual Versus Physical Spaces: Which Facilitates Greater HIV Risk Taking Among Men Who Have Sex with Men in East and South-East Asia?. AIDS Behav.

[19] Williams M, Bowen A, Ei S (2010). An evaluation of the experiences of rural MSM who accessed an online HIV/AIDS health promotion intervention. Health Promot Pract.

[20] Hightow-Weidman LB, Pike E, Fowler B, Matthews DM, Kibe J, McCoy R, Adimora AA (2012). HealthMpowerment.org: feasibility and acceptability of delivering an internet intervention to young Black men who have sex with men. AIDS Care.

[21] Williams ML, Bowen AM, Horvath KJ (2005). The social/sexual environment of gay men residing in a rural frontier state: implications for the development of HIV prevention programs. J Rural Health.

[22] Lyles CM, Crepaz N, Herbst JH, Kay LS, HIV/AIDS Prevention Research Synthesis Team (2006). Evidence-based HIV behavioral prevention from the perspective of the CDC's HIV/AIDS Prevention Research Synthesis Team. AIDS Educ Prev.

[23] Flores SA, Crepaz N, HIV Prevention Research Synthesis Team (2004). Quality of study methods in individual- and group-level HIV intervention research: critical reporting elements. AIDS Educ Prev.

[24] Reback CJ, Grant DL, Fletcher JB, Branson CM, Shoptaw S, Bowers JR, Charania M, Mansergh G (2012). Text messaging reduces HIV risk behaviors among methamphetamine-using men who have sex with men. AIDS Behav.

[25] Hirshfield S, Chiasson MA, Joseph H, Scheinmann R, Johnson WD, Remien RH, Shaw FS, Emmons R, Yu G, Margolis AD (2012). An online randomized controlled trial evaluating HIV prevention digital media interventions for men who have sex with men. PLoS One.

[26] Bourne C, Knight V, Guy R, Wand H, Lu H, McNulty A (2011). Short message service reminder intervention doubles sexually transmitted infection/HIV re-testing rates among men who have sex with men. Sex Transm Infect.

[27] Ko NY, Hsieh CH, Wang MC, Lee C, Chen CL, Chung AC, Hsu ST (2013). Effects of Internet popular opinion leaders (iPOL) among Internet-using men who have sex with men. J Med Internet Res.

[28] Lau JT, Lau M, Cheung A, Tsui HY (2008). A randomized controlled study to evaluate the efficacy of an Internet-based intervention in reducing HIV risk behaviors among men who have sex with men in Hong Kong. AIDS Care.

[29] Rhodes SD, Vissman AT, Stowers J, Miller C, McCoy TP, Hergenrather KC, Wilkin AM, Reece M, Bachmann LH, Ore A, Ross MW, Hendrix E, Eng E (2011). A CBPR partnership increases HIV testing among men who have sex with men (MSM): outcome findings from a pilot test of the CyBER/testing internet intervention. Health Educ Behav.

[30] Bowen AM, Williams ML, Daniel CM, Clayton S (2008). Internet based HIV prevention research targeting rural MSM: feasibility, acceptability, and preliminary efficacy. J Behav Med.

[31] Blas MM, Alva IE, Carcamo CP, Cabello R, Goodreau SM, Kimball AM, Kurth AE (2010). Effect of an online video-based intervention to increase HIV testing in men who have sex with men in Peru. PLoS One.

[32] Carpenter KM, Stoner SA, Mikko AN, Dhanak LP, Parsons JT (2010). Efficacy of a web-based intervention to reduce sexual risk in men who have sex with men. AIDS Behav.

[33] Christensen JL, Miller LC, Appleby PR, Corsbie-Massay C, Godoy CG, Marsella SC, Read SJ (2013). Reducing shame in a game that predicts HIV risk reduction for young adult MSM: a randomized trial delivered nationally over the Web. J Int AIDS Soc.

[34] Mustanski B, Garofalo R, Monahan C, Gratzer B, Andrews R (2013). Feasibility, acceptability, and preliminary efficacy of an online HIV prevention program for diverse young men who have sex with men: the keep it up! intervention. AIDS Behav.

[35] Rosser BR, Oakes JM, Konstan J, Hooper S, Horvath KJ, Danilenko GP, Nygaard KE, Smolenski DJ (2010). Reducing HIV risk behavior of men who have sex with men through persuasive computing: results of the Men's INTernet Study-II. AIDS.

[36] Young SD, Cumberland WG, Lee SJ, Jaganath D, Szekeres G, Coates T (2013). Social networking technologies as an emerging tool for HIV prevention: a cluster randomized trial. Ann Intern Med.

[37] HIV Big Deal.

[38] Ahlberg T (2006). Meth.

[39] Carpenter K HotandSafeM4M.

[40] Branson B, Handsfield HH, Lampe MA, Janssen RS, Taylor AW, Lyss SB, Clark JE, Centers for Disease Control and Prevention (CDC) (2006). Revised recommendations for HIV testing of adults, adolescents, and pregnant women in health-care settings. MMWR Recomm Rep.

